# Aortic Valve Replacement in Asymptomatic Severe Aortic Stenosis: A Systematic Review and Meta-Analysis

**DOI:** 10.1016/j.jscai.2025.103663

**Published:** 2025-05-02

**Authors:** Philippe Généreux, Marko Banovic, Duk-Hyun Kang, Gennaro Giustino, Bernard D. Prendergast, Brian R. Lindman, David E. Newby, Philippe Pibarot, Björn Redfors, Allan Schwartz, Roxanna Seyedin, Bernard Iung, Marc R. Dweck

**Affiliations:** a Gagnon Cardiovascular Institute, Morristown Medical Center, Morristown, New Jersey; b Belgrade Medical School, University of Belgrade, Serbia; c Cardiology Department, University Clinical Center of Serbia, Belgrade, Serbia; d Asan Medical Center, College of Medicine, University of Ulsan, Seoul, Korea; e Department of Cardiology, Guys and St Thomas' NHS Foundation Trust Hospital London, London, United Kingdom; f Heart, Vascular & Thoracic Institute, Cleveland Clinic London, London, United Kingdom; g Structural Heart and Valve Center, Vanderbilt University Medical Center, Nashville, Tennessee; h British Heart Foundation Centre for Cardiovascular Science, University of Edinburgh, Edinburgh, United Kingdom; i Quebec Heart & Lung Institute, Laval University, Quebec City, Quebec, Canada; j Department of Molecular and Clinical Medicine, Institute of Medicine, Gothenburg University, Gothenburg, Sweden; k Department of Cardiology, Sahlgrenska University Hospital, Gothenburg, Sweden; l Department of Population Health Sciences, Weill Cornell Medicine, New York, New York; m NewYork-Presbyterian Hospital/Columbia University Irving Medical Center, New York, New York; n Edwards Lifesciences, Irvine, California; o Bichat Hospital, APHP, and INSERM LVTS 1148, Université Paris-Cité, Paris, France

**Keywords:** aortic stenosis, aortic valve replacement, meta-analysis, systematic review, transcatheter aortic valve replacement

## Abstract

**Background:**

Current guidelines recommend a strategy of clinical surveillance (CS) for patients with asymptomatic severe aortic stenosis and normal left ventricular ejection fraction.

**Methods:**

PubMed, Embase, and ClinicalTrials.gov were searched through November 2024 for randomized controlled trials (RCTs) and observational studies comparing surgical aortic valve replacement or transcatheter aortic valve replacement with CS in patients with asymptomatic severe aortic stenosis.

**Results:**

Sixteen eligible studies (12 observational studies and 4 RCTs) were identified, with a total of 3919 patients in the observational studies and 1427 patients in the RCTs. In the pooled analyses combining observational studies and RCTs, aortic valve replacement (AVR) was associated with significantly reduced all-cause mortality (incidence rate ratio [IRR], 0.42; 95% CI, 0.31-0.58; *P* < .01; *I*^*2*^ = 72%), cardiovascular mortality (IRR, 0.46; 95% CI, 0.28-0.78; *P* < .01; *I*^*2*^ = 68%), and unplanned cardiovascular or heart failure (HF)-related hospitalization (IRR, 0.34; 95% CI, 0.21-0.55; *P* < .01; *I*^*2*^ = 50%). In 12 observational studies, AVR was associated with significantly lower rates of all-cause mortality (IRR, 0.36; 95% CI, 0.27-0.49; *P* < .01; *I*^*2*^ = 65%), and cardiovascular mortality (IRR, 0.33; 95% CI, 0.16-0.70; *P* < .01; *I*^*2*^ = 71%) compared with CS. In 4 RCTs, there was no significant difference in all-cause or cardiovascular mortality, but AVR was associated with a significant reduction in unplanned cardiovascular or HF hospitalization (IRR, 0.42; 95% CI, 0.26-0.65; *P* < .01; *I*^*2*^ = 27%) and stroke (IRR, 0.63; 95% CI, 0.40-0.98*; P* = .04; *I*^*2*^ = 0%).

**Conclusions:**

Data from observational studies and recent RCTs suggest that a strategy of preemptive AVR is associated with improved survival and lower rates of unplanned cardiovascular or HF-related hospitalizations and stroke compared to CS.

## Introduction

Aortic stenosis (AS) is the most prevalent valvular heart disease in developed countries and is often asymptomatic in its early stages, leading to delayed treatment and increased risk of complications.^1^, ^2^, ^3^ Current guidelines do not recommend aortic valve replacement (AVR) in severe AS until the development of symptoms or reduced left ventricular ejection fraction, but rely largely on nonrandomized data and expert opinion.^4^^,^^5^ In addition, the assessment of symptoms related to severe AS is challenging, particularly in older patients.^6^

Several observational studies and randomized trials have assessed the impact of AVR versus clinical surveillance (CS) in patients with asymptomatic severe AS and have shown reductions in all-cause mortality and heart failure (HF) hospitalization following AVR.^6^, ^7^, ^8^, ^9^, ^10^, ^11^, ^12^, ^13^, ^14^, ^15^, ^16^, ^17^, ^18^, ^19^, ^20^, ^21^, ^22^, ^23^, ^24^, ^25^, ^26^ Most recently, 2 randomized controlled trials (RCTs) have evaluated the effects of timely intervention with both transcatheter AVR (TAVR) and surgical AVR (SAVR).^27^^,^^28^ Given these new data, we performed an updated meta-analysis of RCTs and observational studies to characterize the totality of the evidence evaluating AVR (TAVR or SAVR) vs routine CS in this setting (Central Illustration).

**Central Illustration:**
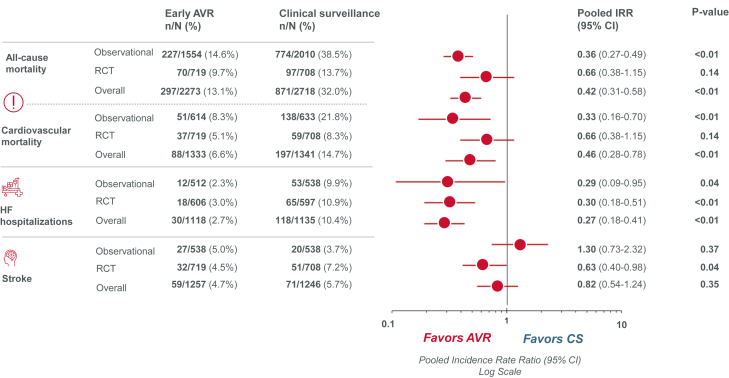
Systematic review and study-level meta-analysis of aortic valve replacement (AVR) vs clinical surveillance (CS) in asymptomatic severe aortic stenosis including 4 randomized trials and 12 observational studies. Among patients with asymptomatic severe aortic stenosis, meta-analysis of the 4 randomized trials showed that early AVR was associated with a lower risk for heart failure (HF) hospitalizations and stroke compared with CS. There were no significant differences in all-cause or cardiovascular mortality between groups. The meta-analysis of the 12 observational studies showed that early AVR was associated with lower risk for all-cause and cardiovascular mortality, and HF hospitalizations with CS, with no difference in stroke. IRR, incidence rate ratio; RCT, randomized controlled trial.

## Methods

### Eligibility criteria, databases, and search strategy

We conducted a systematic literature review based on the Cochrane Handbook for Systematic Reviews of Interventions.^29^ Following this review, a meta-analysis was performed according to the Preferred Reporting Items for Systematic Reviews and Meta-Analyses (PRISMA) 2020 statement.^30^ The Population, Intervention, Comparator, Outcomes, and Study (PICOS) design framework was utilized as an eligibility criterion to search, select, and review relevant studies (Supplemental Table S1). We included both RCTs and observational studies if they fulfilled the following criteria: (1) asymptomatic patients with severe or very severe AS treated with AVR (SAVR or TAVR) or conservative CS, and (2) availability of clinical outcome data. Abstracts, review articles, case reports, letters, editorials, and nonjournal literature were excluded.

We searched PubMed, Embase, and ClinicalTrials.gov using prespecified criteria from inception until November 11, 2024. To increase the sensitivity of the search, variants of the phrases “asymptomatic aortic stenosis,” “severe aortic stenosis,” “aortic valve replacement,” “surgical aortic valve replacement,” “intervention,” “conservative treatment,” and “conservative management” were developed as either medical subject heading terms in PubMed, Emtree terms in Embase, and text words related to AVR in asymptomatic severe AS. The search strategy had no restrictions on language, publication date, age, living setting, gender, race, ethnicity, or geographical location of the patient population. Details of the search strategy are presented in Supplemental Table S2.

Two reviewers (P.G. and R.S.) independently screened against predefined eligibility criteria using DistillerSR Version 2.35 (DistillerSR Inc, 2025) in 2 phases: (phase 1) title or abstract screening, and (phase 2) full-text screening. Disputes were resolved by a third independent reviewer (G.G.). This study was registered with the International Platform of Registered Systematic Reviews and Meta-analysis Protocols (registration number: INPLASY202530112).

### Clinical outcomes

The primary outcome assessed was all-cause mortality. Secondary outcomes included cardiovascular mortality, unplanned cardiovascular or HF-related hospitalizations, and stroke.

### Data extraction and risk of bias assessment

Two independent reviewers (R.S. and G.G.) abstracted data on the study population, baseline demographics, interventions, and outcomes of interest using Nested Knowledge (Nested Knowledge, Inc, 2025). Any discrepancies in collected data were resolved by consensus or consultation with a third independent reviewer (P.G.). For instances where studies had multiple sequential publications, we collected the most recent data. All studies were independently assessed by 2 reviewers (R.S. and G.G.) and any disagreements were resolved through discussion with a third reviewer. The risk of bias (RoB) of each RCT was assessed using the Cochrane Risk of Bias 2 tool for RCTs.^31^ The RoB for observational studies was assessed using the Newcastle-Ottawa Scale.^32^

### Statistical analysis

A meta-analysis using the inverse variance method was conducted for outcomes of interest using the “metafor” package (V.4.4-0) from R version 4.0.5 (R Foundation for Statistical Computing).^29^^,^^33^^,^^34^ Event rates were standardized to incidence rate ratios (IRR) to account for differences in the overall duration of follow-up across studies and between treatment arms within studies. To derive IRR, total person-time was extracted from included studies or estimated using the median follow-up time of each treatment arm multiplied by each arm’s sample size.^34^ For studies that reported a mean follow-up time, the median follow-up was estimated assuming an exponential distribution. To account for patients lost to follow-up, studies reporting mean or median follow-up times were prioritized (selected for inclusion in the primary analysis) over those reporting only total study duration. For all outcomes, pooled IRR and their corresponding 95% CI were calculated using a random-effects model. Heterogeneity was assessed using the Higgins *I*^*2*^ statistic, where low, moderate, substantial, and high heterogeneity were defined as <40%, 30% to 60%, 50% to 90%, and >75%, respectively.^29^ Publication bias was assessed using funnel plots and Egger’s linear-regression test to detect funnel plot asymmetry.^29^^,^^35^

The primary cohort analysis was conducted using only studies that reported either a mean or median follow-up time, with subgroup analyses performed according to study design (ie, RCTs versus observational studies) for all outcomes of interest. All subgroups were tested for statistical interaction using the χ^2^ statistic. We also performed a sensitivity analysis of the primary outcome, including all studies that reported either full study duration or a mean or median follow-up time. This article does not report on patients or patient-level data.

## Results

### Study selection and characteristics

**Figure 1:**
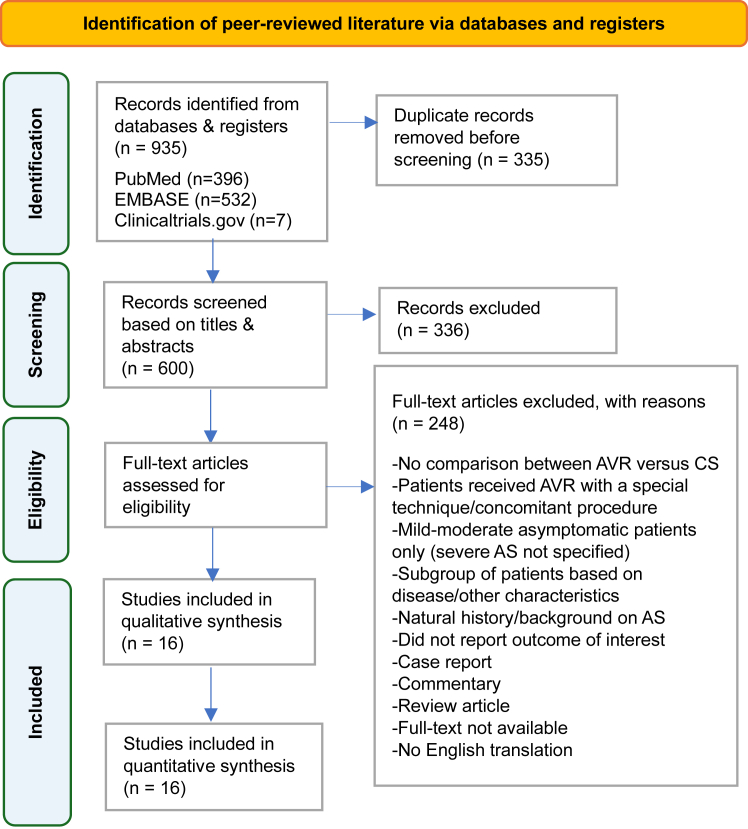
**Preferred Reporting Items for Systematic Reviews and Meta-analysis (PRISMA) flow diagram.** AS, aortic stenosis; AVR, aortic valve replacement; CS, clinical surveillance.

The search identified a total of 935 records, from which 335 duplicates and 336 additional irrelevant titles and abstracts were excluded. The remaining 264 studies were subject to full-text screening. Sixteen were deemed eligible for inclusion in the qualitative and quantitative syntheses (Figure 1)—12 observational cohort studies (4 prospective, 8 retrospective)^7^, ^8^, ^9^, ^10^, ^11^, ^12^, ^13^, ^14^, ^15^, ^16^, ^17^, ^18^ and 4 RCTs (Table 1).^25^, ^26^, ^27^, ^28^ Key baseline and study characteristics are summarized in Table 2,^7^, ^8^, ^9^, ^10^, ^11^, ^12^, ^13^, ^14^, ^15^, ^16^, ^17^, ^18^^,^^25^, ^26^, ^27^, ^28^ with additional features of the included trials in Table 1.

**Table 1:** Summary of randomized controlled trials.

	Généreux et al,^27^ 2025 EARLY TAVR	Loganath et al,^28^ 2025 EVOLVED	Banovic et al,^26^ 2024 AVATAR	Kang et al,^25^ 2020 RECOVERY
Inclusion criteria	Age ≥ 65 y; AVA ≤ 1 cm^2^ or iAVA ≤ 0.6 cm^2^/m^2^ and (V_max_ ≥ 4.0 m/s or MG ≥ 40 mm Hg); asymptomatic (confirmed exercise testing); STS score ≤ 10; LVEF ≥ 50%; low level stress test (90.6%)	Age ≥ 18 y; V_max_ ≥ 4.0 m/s or (iAVA < 0.6 cm^2^/m^2^ ​and V_max_ ≥ 3.5 m/s); midwall LGE on CMR; no symptoms attributable to AS that require AVR; LVEF ≥ 50%; no stress test reported	Age ≥18 y; (AVA ≤ 1 cm^2^ or iAVA ≤ 0.6 cm^2^/m^2^ at rest) and ​(V_max_ > 4.0 m/s or MAG ≥ 40 mm Hg); without reported symptoms; STS score < 8%; LVEF ≥ 50%; low level stress test (100%)	Age 20-80 y; AVA ≤ 0.75 cm^2^ and (V_max_ ≥ 4.5 m/s or MAG ≥ 50 mm Hg); asymptomatic; candidate for early surgery; LVEF ≥ 50%; low level stress test (17%)
No. of patients	901	224	157	145
Mean age, y	75.8	73.4	67.0	64.5
Bicuspid aortic valve; female patients (% of patients)	8%; 31%	29%; 28%	14%; 43%	61%; 51%
AVA, cm^2^^a^	TAVR: 0.9 ± 0.23 CS: 0.8 ± 0.23	SAVR/TAVR: 0.8 ± 0.2 CS: 0.8 ± 0.2	SAVR: 0.73 (0.55, 0.84) CS: 0.74 (0.59, 0.89)	SAVR: 0.63 ± 0.09 CS: 0.64 ± 0.09
Vmax, m/s^a^	TAVR: 4.3 ± 0.45 CS: 4.4 ± 0.43	SAVR/TAVR: 4.3 ± 0.5 CS: 4.4 ± 0.5	SAVR: 4.5 (4.3, 4.8) CS: 4.5 (4.2, 4.7)	SAVR: 5.14 ± 0.52 CS: 5.04 ± 0.44
Pmean, mm Hg^a^	TAVR: 46.5 ± 10.08 CS: 47.3 ± 10.61	SAVR/TAVR: 45.2 ± 11.5 CS: 45.0 ± 10.2	SAVR: 51 (44, 58) CS: 50 (43, 59)	SAVR: 64.3 ± 14.4 CS: 62.7 ± 12.4
Median (IQR) follow-up	TAVR: 3.7 (3.0, 5.1) y CS: 3.8 (2.8, 4.8) y	TAVR/SAVR: 4.0 (1.0, 4.3) y CS: 3.0 (1.1, 4.1) y	SAVR: 63 (48, 75) mo CS: 63 (48, 75) mo	SAVR: 6.2 (5.0, 7.4) y CS: 6.1 (4.5, 7.3) y
Median (IQR) time to AVR	14 (9, 24) days to TAVR	152.1 (103.4, 243.3) days to SAVR/TAVR	55 (36, 79) days to SAVR	23 (10, 36) days to SAVR
Median (IQR) time AVR from randomization (CS group)	11.1 (5.0, 19.7) mo to TAVR	614.4 (346.8, 1277.5) days to SAVR/TAVR	476 (226, 1098) days to SAVR	700 (277, 1469) days to SAVR
Median (IQR) time to conversion to AVR upon symptom development/indication for AVR (CS group)	32 (18, 58) days to TAVR	100 (43,146) days to SAVR/TAVR	123 (90, 297) days to SAVR	Not reported
Key findings	· Met primary end point (superiority)^b^ · Significantly lower incidence of the composite end point in the early TAVR arm compared with the CS arm (26.8% vs 45.3%; HR, 0.50; 95% CI, 0.40-0.63; *P <* .0001) · No difference in mortality	· Did not meet primary end point^b^ · Significantly lower incidence of AS-related hospitalizations in the AVR arm compared with the CS arm (6.2% vs 17.1%; HR, 0.37; 95% CI, 0.16-0.88; *P =* .024) · No difference in mortality	· Met primary end point (superiority)^b^ · Significantly lower incidence of the composite end point in SAVR compared with the CS arm (23.1% vs 46.8%; HR, 0.42; 95% CI, 0.24-0.73; *P =* .002) · Lower rate of mortality with SAVR	· Met primary end point (superiority)^b^ · Significantly lower incidence of the composite end point in SAVR compared with the CS arm (1% vs 15%; HR, 0.09; 95% CI, 0.01-0.67; *P =* .003) · Lower rate of mortality with SAVR

AS, aortic stenosis; AVR, aortic valve replacement; CMR, cardiac magnetic resonance; CS, clinical surveillance; HR, hazard ratio; iAVA, indexed aortic valve area; LGE, late gadolinium enhancement; LVEF, left ventricular ejection fraction; MAG, mean aortic valve gradient; Pmean, mean transaortic valvular gradient; SAVR, surgical aortic valve replacement; SD, standard deviation; STS, Society of Thoracic Surgeons; TAVR, transcatheter aortic valve replacement; Vmax, maximal systolic aortic flow velocity; vs, versus.

a Reported as median (IQR) or mean ± SD at baseline.

b Primary end point for the following: (1) EARLY TAVR: all-cause death, all stroke, and unplanned cardiovascular hospitalization when all patients have reached 2-year follow-up; (2) EVoLVeD: composite of all-cause mortality or unplanned AS-related hospitalization from randomization through study completion (mean follow-up expected to be an average of 2.75 years); (3) AVATAR: all-cause mortality or major adverse cardiovascular events composed of acute myocardial infarction, stroke, and unplanned heart failure hospitalization needing intravenous treatment within 5-year follow-up; (4) RECOVERY: operative mortality (during or within 30 days of surgery) or cardiac mortality during entire follow-up (a minimum of 4 years).

**Table 2:** Key study and baseline characteristics.

Reference, year	Country	Study period	No. of patients			Mean age, y	Follow-up	Time to AVR	LVEF criteria	Stress test (% patients)	AS severity
			Total	AVR	CS						
Randomized controlled trials											
Généreux et al,^27^ 2025 EARLY TAVR	US and CAN	2017-2021	901	TAVR: 455	446	75.8	Median AVR: 3.7 y CS: 3.8 y	Median 14 d	Yes (≥50%)	Yes (90.6%)	AVA ≤ 1 cm^2^ or iAVA ≤ 0.6 cm^2^/m^2^ and Vmax ≥ 4.0 m/s or MAG ≥ 40 mm Hg
Loganath et al,^28^ 2025 EVOLVED	UK	2017-2022	224	SAVR/TAVR: 113	111	73.4	Median AVR: 4.0 y CS: 3.0 y	Median 152.1 d	Yes (≥50%)	Not reported	Vmax ≥ 4.0 m/s or iAVA < 0.6 cm^2^/m^2^ ​and Vmax ≥ 3.5 m/s
Banovic et al,^26^ 2024 AVATAR	Europe	2015-2023	157	SAVR: 78	79	67.0	Median AVR: 63 mo CS: 63 mo	Median 55 d	Yes (≥50%)	Yes (100%)	AVA ≤ 1 cm^2^ or iAVA ≤ 0.6 cm^2^/m^2^ ​at rest and Vmax > 4.0 m/s or MAG ≥ 40 mm Hg
Kang et al,^25^ 2020 RECOVERY	KOR	2010-2015	145	SAVR: 73	72	64.5	Median AVR: 6.2 y CS: 6.1 y	Median 23 d	Yes (≥50%)	Yes (17%)	AVA ≤ 0.75 cm^2^ and Vmax ≥ 4.5 m/s or MAG ≥ 50 mm Hg
Observational studies											
Çelik et al,^18^ 2021	NLD	2006-2009	8	AVR: 3	5	68.8	Mean 106.8 mo	NA	Yes (≥50%)	Yes (79.7%)	AVA ≤ 1 cm^2^ or Vmax ≥ 4.0 m/s
Campo et al,^17^ 2019	US	2005-2013	265	104	161	70.6	Study length 60.0 mo	AVR within 60 d	None	Yes (30%)	AVA ≤ 1 cm^2^ or Vmax ≥ 4.0 m/s or MAG ≥40 mm Hg
Kim et al,^16^ 2019	KOR	2000-2015	468	SAVR: 221	247	64.2	Median 60.9 mo; PY 2755	Median 49 d	Yes (≥50%)	No	AVA ≤1 cm^2^ or iAVA ≤0.6 cm^2^/m^2^ ​or Vmax ≥4.0 m/s or MAG ≥40 mm Hg
Bohbot et al,^15^ 2018	BEL and FRA	2000-2015	439	SAVR: 192	247	73.0	Median 42.0 mo	Mean 51 d	Yes (≥50%)	Yes (64%)	MAG ≥40 mm Hg
Oterhals et al,^14^ 2017	NOR	2013	31	AVR: 5 TAVR: 2	24^a^	79.0	Study length 18.0 mo	NA	Yes (≥50%)	Yes (15%)	AVA <1 cm^2^ or Vmax >4.0 m/s or MAG >40 mm Hg
Masri et al,^13^ 2016	US	2001-2012	533	SAVR: 341	192	66.0	Mean 82.8 mo	NA	Yes (≥50%)	Yes (100%)	iAVA ≤0.6 cm^2^/m^2^
Taniguchi et al,^12^ 2015	JPN	2003-2011	582	291	291	72.4	Median 44.7 mo	Median 44 d	None	No	AVA <1 cm^2^ or Vmax > 4.0 m/s or MAG > 40 mm Hg
Heuvelman et al,^11^ 2012	NLD	2006-2009	59	22	37	69.9	Study length 24.0 mo	NA	None	Yes (79.6%)	AVA ≤ 1 cm^2^ or Vmax ≥ 4.0 m/s
Le Tourneau et al,^10^ 2010	US	1984-1995	674	SAVR: 160	514	71.0	Average >60 mo; PY 3817	NA	None	No	Vmax ≥ 4.0 m/s
Kang et al,^9^ 2010	KOR	1996-2006	197	SAVR: 102	95	63.0	Median AVR: 41.6 mo CS: 58.2 mo	SAVR within 90 days echo evaluation	Yes (≥50%)	No	AVA ≤ 0.75 cm^2^ and Vmax ≥ 4.5 m/s or MAG ≥ 50 mm Hg
Pai et al,^8^ 2006	US	1993-2003	338	SAVR: 99	239	70.0	Mean 42.0 mo	NA	None	No	AVA ≤ 0.8 cm^2^
Pellikka et al,^7^ 2005	US	1984-1995	325	SAVR: 145	180	72.0	Mean 64.8 mo	SAVR within 90 days of dx	None	No	Vmax ≥ 4.0 m/s

AS, aortic stenosis; AVA, aortic valve area; AVR, aortic valve replacement; BEL, Belgium; CAN, Canada; CS, clinical surveillance; dx, diagnosis; echo: echocardiogram; FRA, France; iAVA, indexed aortic valve area; JPN, Japan; KOR, Korea; LVEF, left ventricular ejection fraction; MAG, mean aortic valve gradient; mo, months; NA, not available; NLD, Netherlands; NOR, Norway; PY, patient-years; SAVR, surgical aortic valve; replacement; TAVR, transcatheter aortic valve replacement; UK, United Kingdom; US, United States; Vmax, maximal systolic aortic flow velocity.

a Thirteen of 24 patients in the CS group had severe AS and 11 had moderate AS.

The final quantitative analysis included a total of 5346 patients (2406 patients in the AVR group and 2940 in the CS group; 1427 patients in RCTs and 3919 in observational studies) with a weighted mean per-patient follow-up of 55.5 months across all studies (49.8 months in RCTs and 57.5 months in observational studies). The follow-up duration ranged from a total study length of 18 months to a population mean follow-up of 106.8 months across all studies. The mean age of patients weighted across all the included studies was 70.6 years (range 63-79 years)—4 RCTs 73.3 years (range 64.5-75.8 years)^25^, ^26^, ^27^, ^28^; 12 observational studies 69.5 years (range 63-79 years).^7^, ^8^, ^9^, ^10^, ^11^, ^12^, ^13^, ^14^, ^15^, ^16^, ^17^, ^18^

The 16 studies included 25,131 patient-years of follow-up data (AVR group 11,582 patient-years, CS group 13,548 patient-years).^7^, ^8^, ^9^, ^10^, ^11^, ^12^, ^13^, ^14^, ^15^, ^16^, ^17^, ^18^^,^^25^, ^26^, ^27^, ^28^ There were 5878 patient-years of follow-up data available across the 4 RCTs,^25^, ^26^, ^27^, ^28^ and 17,763 patient-years across the 12 observational studies.^7^, ^8^, ^9^, ^10^, ^11^, ^12^, ^13^, ^14^, ^15^, ^16^, ^17^, ^18^^,^^25^, ^26^, ^27^, ^28^

### Clinical outcomes

**Figure 2:**
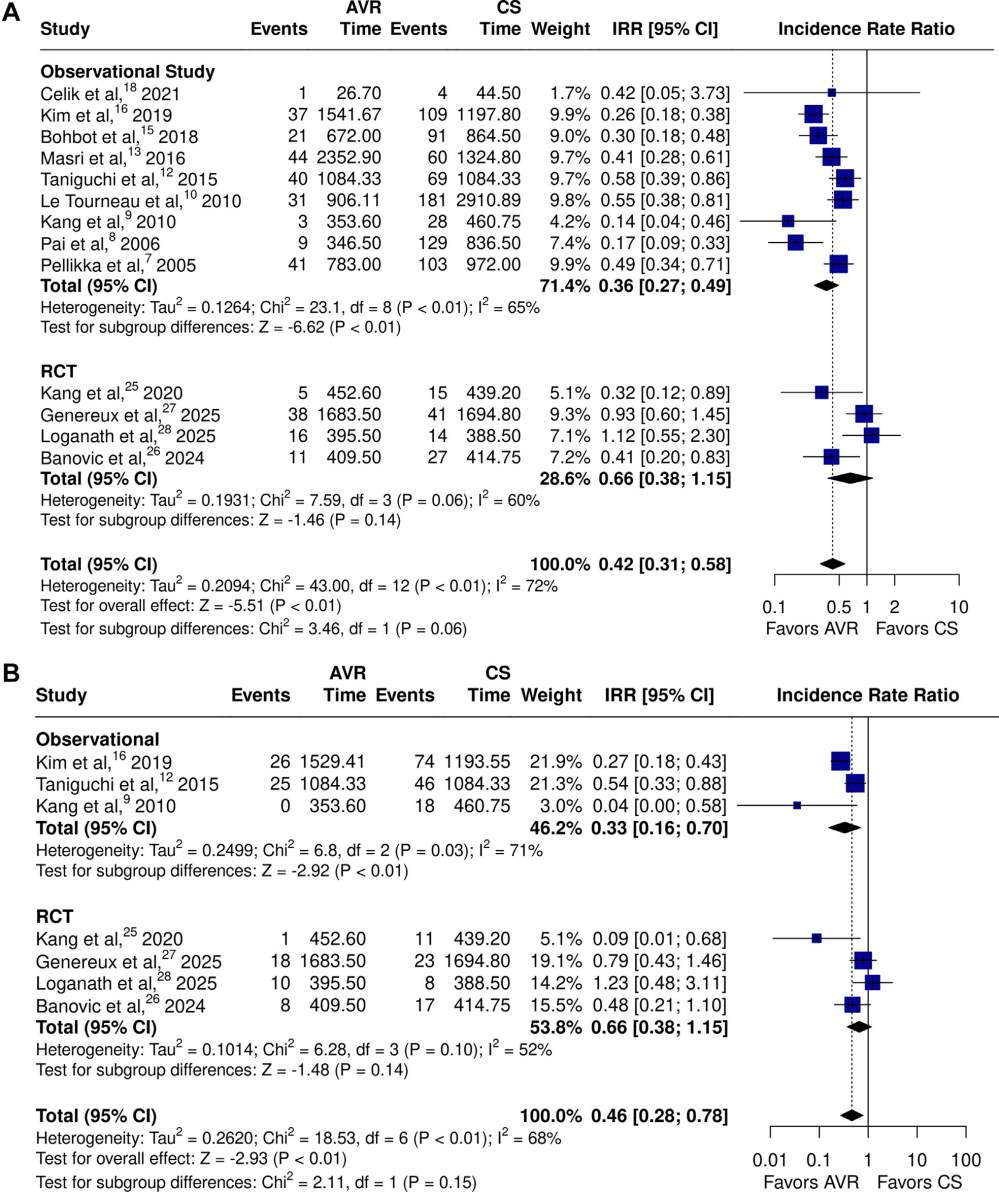
**Meta-analysis of aortic valve replacement (AVR) versus clinical surveillance (CS) comparing incidence rates of (A) all-cause mortality and (B) cardiovascular mortality.** Forest plots with incidence rate ratios (IRR) for (**A**) all-cause mortality^7^, ^8^, ^9^, ^10^^,^^12^^,^^13^^,^^15^^,^^16^^,^^18^^,^^25^, ^26^, ^27^, ^28^ and (**B**) cardiovascular mortality comparing AVR versus CS in randomized controlled trials (RCTs) and observational studies.^9^^,^^12^^,^^16^^,^^25^, ^26^, ^27^, ^28^

In the primary cohort analysis of observational studies and RCTs reporting mean or median follow-up time (13/16 studies), AVR was associated with significantly lower rates of all-cause mortality compared to CS with very high heterogeneity (Figure 2A^7^, ^8^, ^9^, ^10^^,^^12^^,^^13^^,^^15^^,^^16^^,^^18^^,^^25^, ^26^, ^27^, ^28^; IRR, 0.42; 95% CI, 0.31-0.58; *P* < .01; *I*^*2*^ = 72%). This difference was largely driven by observational studies (IRR, 0.36; 95% CI, 0.27-0.49; *P* < .01; *I*^*2*^ = 65%; total patient-years = 17,763, total patients = 3564, average follow-up = 4.98 years), whereas no significant differences in mortality were observed in RCTs (IRR, 0.66; 95% CI: 0.38-1.15; *P* = .14; *I*^*2*^ = 60%; total patient-years = 5878, total patients = 1427, average follow-up = 4.12 years). In the 7 studies reporting rates of cardiovascular mortality, AVR was also associated with significantly lower cardiovascular mortality compared to CS with high heterogeneity (Figure 2B,^9^^,^^12^^,^^16^^,^^25^, ^26^, ^27^, ^28^; IRR, 0.46; 95% CI, 0.28-0.78; *P* < .01; *I*^*2*^ = 68%). Again, this difference was largely driven by observational studies (IRR, 0.33; 95% CI, 0.16-0.70; *P* < .01; *I*^*2*^ = 71%*;* total patient-years = 5706, total patients = 1247, average follow-up = 4.58 years), whereas RCTs alone showed similar rates of cardiovascular mortality (IRR, 0.66; 95% CI: 0.38-1.15; *P* = .14; *I*^*2*^ = 52%*;* total patient-years = 5878, total patients = 1427, average follow-up = 4.12 years).

**Figure 3:**
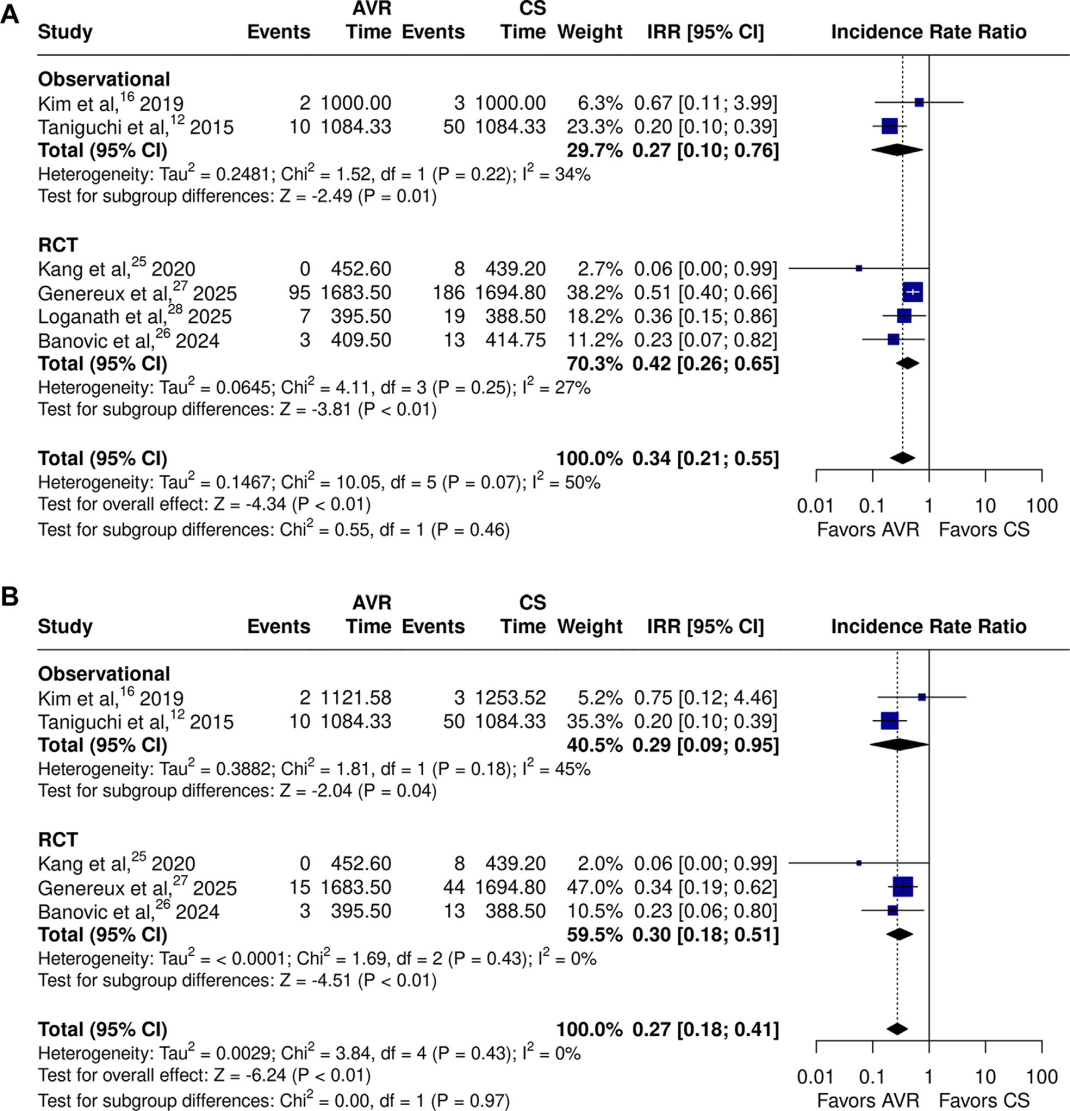

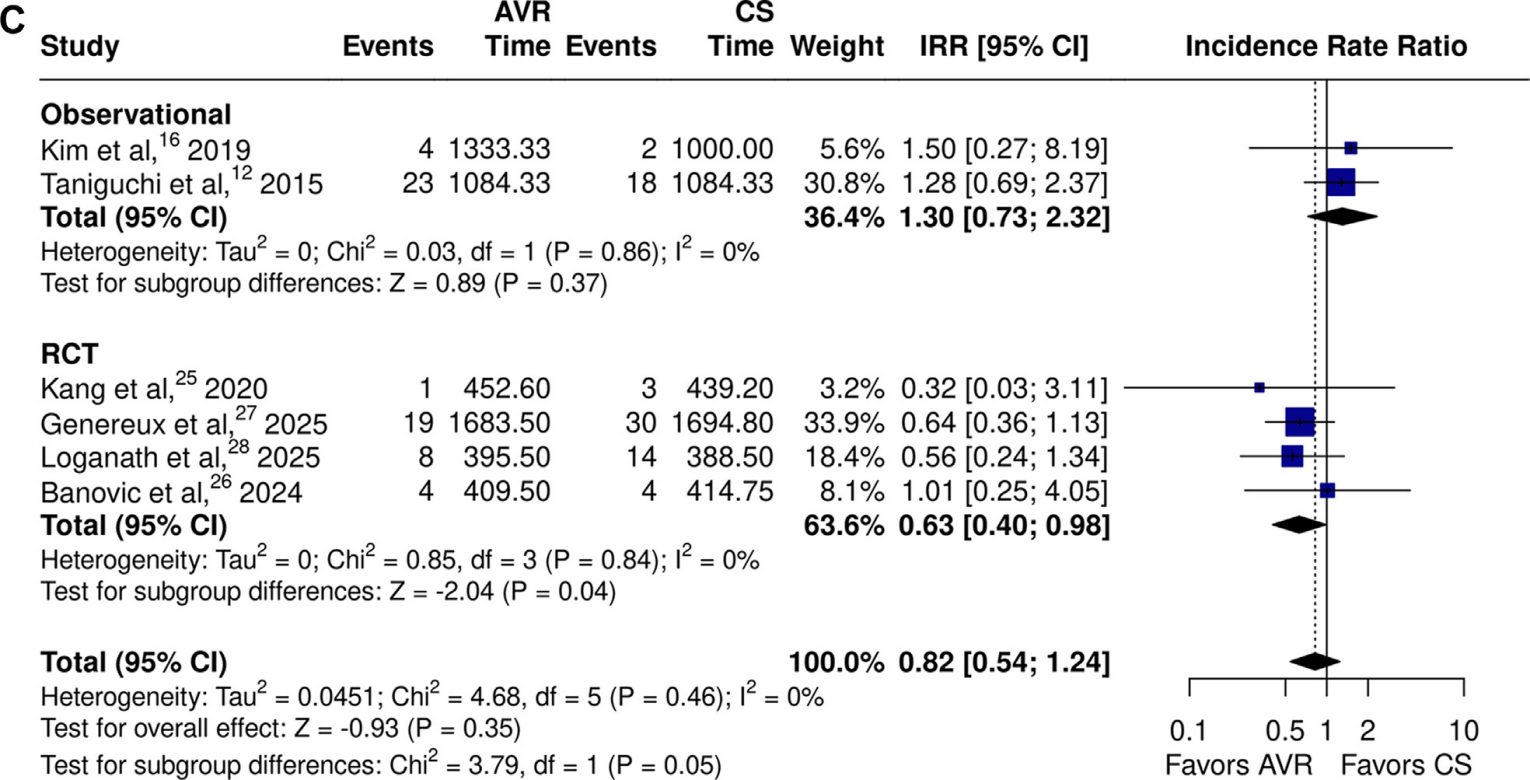
**Meta-analysis of aortic valve replacement (AVR) versus clinical surveillance (CS) comparing incidence rates of (A) unplanned cardiovascular or heart failure (HF) hospitalization^^a,b^^, (B) HF hospitalization, and (C) stroke.** Forest plots with incidence rate ratio (IRR) for (**A**) unplanned cardiovascular or HF hospitalization,^12^^,^^16^^,^^25^, ^26^, ^27^, ^28^ (**B**) HF hospitalization,^12^^,^^16^^,^^25^, ^26^, ^27^**(C**) and stroke comparing AVR versus CS in randomized controlled trials (RCTs) and observational studies.^12^^,^^16^^,^^25^, ^26^, ^27^, ^28^^a^Unplanned cardiovascular hospitalization in EARLY TAVR (Généreux et al,^27^ 2025) is defined as admission through the emergency department or same-day admission from a clinic for congestive heart failure or aortic stenosis (AS)-related causes, as well as other cardiovascular causes like arrhythmia/conduction system disturbance, bleeding, coronary artery disease, stroke/transient ischemic attack, thromboembolic event, and any aortic valve intervention within 6 months of randomization in the CS arm, including conversion to AVR, and any aortic valve reintervention within 6 months of the procedure in the TAVR arm. ^b^Unplanned AS hospitalization in EVOLVED (Loganath et al,^28^ 2025) is defined as any unplanned admission before or after aortic valve replacement with syncope, heart failure, chest pain, ventricular arrhythmia or second- or third-degree heart block, attributed to aortic valve disease.

Regarding secondary outcomes, analysis of 6 studies showed that AVR was associated with significantly lower rates of unplanned cardiovascular or HF-related hospitalization with moderate heterogeneity (Figure 3A^12^^,^^16^^,^^25^, ^26^, ^27^, ^28^; IRR, 0.34; 95% CI, 0.21-0.55; *P* < .01; *I*^*2*^ = 50%). When considered separately, both observational studies (IRR, 0.27; 95% CI: 0.10-0.76; *P* = .01; *I*^*2*^ = 34%; total patient-years = 4502, total patients = 1050, average follow-up = 4.29 years) and RCTs (IRR, 0.42; 95% CI: 0.26-0.65; *P* < .01; *I*^*2*^ = 27%; total patient-years = 5878, total patients = 1427, average follow-up = 4.12 years) demonstrated that timely AVR was associated with significantly lower rates of unplanned cardiovascular or HF-related hospitalization, with low heterogeneity. Similar findings were observed for the incidence of HF hospitalization across observational studies and RCTs (Figure 3B).^12^^,^^16^^,^^25^, ^26^, ^27^

Although there were no significant differences in stroke overall (Figure 3C,^12^^,^^16^^,^^25^, ^26^, ^27^, ^28^; IRR, 0.82; 95% CI, 0.54-1.24; *P* = .35; *I*^*2*^ = 0%), there were significant reductions associated with early intervention in RCTs (IRR, 0.63; 95% CI: 0.40-0.98; *P =* .04; *I*^*2*^ = 0%; total patient-years = 5878, total patients = 1427, average follow-up = 4.12 years), but not in the observational studies (IRR, 1.30; 95% CI, 0.73-2.32; *P =* .37; *I*^*2*^ = 0%; total patient-years = 4502, total patients = 1050, average follow-up = 4.29 years).

Subgroup analyses indicated no significant differences between RCTs and observational studies for almost all outcomes. The only exception was stroke which was less frequent among patients undergoing timely AVR in the majority of RCTs, with opposite trends in observational studies (Figure 3C; χ^2^ = 3.79; *P*_*interaction*_ = .05). The sensitivity analysis including all 16 studies showed similar results for all-cause mortality as the primary cohort analysis (Supplemental Figure S1; IRR, 0.44; 95% CI, 0.33-0.59; *P* < .01; *I*^*2*^ = 68%).

### RoB and publication bias

Overall RoB bias was considered “low” among the individual RCTs (Supplemental Table S3). Similarly, RoB was assessed for the 12 observational studies and the quality of these data was good as depicted on the Newcastle-Ottawa Scale for observational studies (Supplemental Table S4). The overall ratings across studies ranged between 5 and 8 points, with 8 studies rated at 8 points and 3 studies receiving an overall rating of 7 points. One study was rated at 6 points and had a low rating for comparability, as very little detail was provided for patient baseline characteristics by AVR and CS groups.^15^ Assessed visually, the funnel plots showed no evident systematic bias (Supplemental Figure S2). Similarly, no publication bias was detected for the primary analysis of all-cause mortality using Egger’s test (*P* = .53). Publication bias for other outcomes could not be statistically assessed, as tests for asymmetry have low power when fewer than 10 studies are available.

## Discussion

This is the largest meta-analysis to characterize the breadth of evidence assessing the effects of AVR compared with CS in patients with asymptomatic severe AS. We synthesized data from 16 studies, including 5346 patients with asymptomatic severe AS (left ventricular ejection fraction ≥50%), 2406 of whom underwent AVR and 2940 who were managed with conservative CS. Results from the pooled primary cohort analysis demonstrated that AVR was associated with significant reductions in the rates of all-cause mortality, cardiovascular mortality, and unplanned cardiovascular or HF-related hospitalization compared to CS, with low to moderate heterogeneity across all outcomes except all-cause and cardiovascular mortality. Subgroup analyses showed that AVR was associated with the following: (1) significantly lower rates of all-cause and cardiovascular mortality in observational studies, although this effect was not observed when the analysis was restricted to RCTs alone; (2) significantly lower rates of unplanned cardiovascular or HF-related hospitalization in both observational studies and RCTs; and (3) a significant reduction in stroke rates compared with CS across the 4 randomized trials.

Patients with AS have increased mortality risk across all degrees of severity, and this risk is further exacerbated with the onset of symptoms.^6^^,^^36^, ^37^, ^38^, ^39^, ^40^, ^41^ Although patients with symptomatic severe AS have a guideline-recommended indication for AVR,^5^ the optimal timing of intervention in patients with asymptomatic severe AS remains a matter of active debate. Approximately 50% of patients will become symptomatic and require AVR within 1 year of initial diagnosis, with a mortality risk that increases by ∼1% per annum while asymptomatic, to 4% in the first 3 months after symptom onset.^6^^,^^27^ Furthermore, sustained and increasing pressure overload during CS is also associated with irreversible structural and functional impairment of the left ventricle and other cardiac structures, which are associated with an increased likelihood of HF and death.^27^^,^^36^^,^^38^^,^^42^ TAVR is a less invasive alternative to SAVR that is now established as the standard of care for patients with severe AS across the spectrum of surgical risk.^43^, ^44^, ^45^, ^46^, ^47^, ^48^, ^49^ Over the last decade, evidence for timely AVR has primarily focused on SAVR, with several observational studies and randomized trials providing strong signals that favor SAVR over CS.^8^, ^9^, ^10^^,^^15^, ^16^, ^17^^,^^25^^,^^26^ More recently, the EARLY TAVR trial demonstrated the superiority of a strategy of early TAVR in reducing the incidence of all-cause mortality, stroke, or unplanned cardiovascular hospitalization compared to CS.^27^

Using an exhaustive search of relevant literature evaluating the outcomes of AVR in asymptomatic patients with severe AS, this meta-analysis included more studies compared to previous work to date, notably incorporating recent data from the EARLY TAVR and EVOLVED trials, as well as updated results from the AVATAR trial. Our study is also the first to characterize the strength of evidence of both SAVR and TAVR against a CS strategy in these patients, and to comparatively assess results derived from observational studies^7^, ^8^, ^9^, ^10^, ^11^, ^12^, ^13^, ^14^, ^15^, ^16^, ^17^, ^18^ and randomized trials.^25^, ^26^, ^27^, ^28^ Across observational studies, AVR was associated with significantly lower rates of all-cause and cardiovascular mortality compared with CS, whereas these findings trended in favor of AVR across RCTs, but did not reach statistical significance. These differences are potentially due to selection and treatment bias when comparing patients who underwent AVR versus CS in a nonrandomized fashion, or less rigorous follow-up and cross-over to interventional treatment in the CS arm of observational studies. However, our subgroup analyses showed no differences for all-cause mortality and cardiovascular mortality according to the study design.

Although data from RCTs remain the gold standard for estimating the effects of interventions and observational studies are subject to bias, it is important to recognize that not all observational studies are of equal evidentiary value and that these studies can provide a closer representation of routine practice, the impact of delayed treatment on clinical outcomes, and the realities of undertreatment that may not typically be observed in RCTs.^50^ Multiple studies have demonstrated the detrimental impact of waiting for AVR while symptomatic, especially in geographic regions where prompt access to care is challenging.^51^^,^^52^ In this meta-analysis, we sought to broadly capture the totality of robust evidence evaluating the outcomes of AVR in asymptomatic severe AS patients over time, rather than relying solely on randomized trial data with limited minority trial representation and a lack of practical comparisons to real-world standard of care.

The results of this analysis showed considerable heterogeneity across 3 of the 5 reported outcomes. The heterogeneity in mortality estimates is in part due to differences in the patient populations and the management strategies observed across the included studies, with differing methodologies and clinical settings. Patients enrolled in the surgical trials (RECOVERY and AVATAR) were younger with severe or critical AS, a group more likely to benefit from AVR and undergo a successful SAVR. In contrast, the EARLY TAVR trial enrolled older patients with multiple competing comorbidities, which may have contributed to increased mortality and morbidity in both groups, thereby likely attenuating the beneficial effects of early TAVR. Importantly, despite competing risks related to age and comorbidities, the benefit of prompt intervention was still broadly observed across the majority of studies (albeit less pronounced in some), with patients ranging from 63 to 79 years of age.

Moreover, the quality of CS and the time between the recognition of symptoms to AVR differed substantially across studies. This time interval was prolonged in the surgical trials, with patients in the CS arm often waiting several months before undergoing treatment. These delays suggest that outcome differences between the AVR and CS groups in the surgical trials may have been more pronounced than what was observed in the EARLY TAVR trial. In other words, studies where CS was less optimal demonstrated increased mortality associated with CS that was likely related (at least in part) to prolonged exposure to symptomatic severe or critical AS prior to AVR, thereby exaggerating the differences in prognosis between the 2 groups and underestimating the true benefit of timely AVR.

The longer delays to AVR observed in the surgical trials may be attributed to the reluctance of patients to undergo surgery due to concerns related to procedure-related complications, as well as health care system differences in the countries where these studies were conducted (South Korea, Serbia, and the United Kingdom), which have less timely access to care than in the United States. Indeed, the quality of CS and prompt access to care seem to be major determinants of the presence or absence of benefits associated with timely intervention. Additionally, all patients in the EARLY TAVR trial were sent for a complete pre-TAVR evaluation prior to randomization. As such, upon symptom onset, patients were able to avoid usual health care system delays, resulting in a shorter time window for them to be exposed to uncorrected symptomatic severe AS. It should also be noted that the quality of CS observed in the most recent RCT is not necessarily feasible in routine clinical practice worldwide, providing a likely explanation for the substantial reduction in mortality associated with AVR in observational studies compared to CS.

Our study showed that AVR was associated with a ∼50% reduction in the relative risk of HF-related hospitalizations compared with CS in both observational studies and RCTs.^53^ This is a notable finding, as HF hospitalization is strongly associated with increased mortality during prolonged follow-up, both after AVR and in HF in general.^54^^,^^55^ More importantly, timely treatment of asymptomatic severe AS may allow AVR to be undertaken in stable conditions rather than in an acute setting, thereby reducing the risk of irreversible complications, which may occur if new symptoms are reported too late during CS.

In the analysis restricted to RCTs alone, we also observed a significant reduction in the rate of stroke events favoring AVR when compared with CS. This result may be considered hypothesis-generating because one of the major concerns of timely intervention is the possibility of procedure-related complications such as stroke, major vascular events, or bleeding.^56^, ^57^, ^58^ Potential explanations for this finding include reduced peri-procedural complications related to elective rather than urgent AVR, increased cardiac damage with left atrial enlargement leading to undetected atrial fibrillation, and increased aortic valve calcification leading to microembolism before or during AVR. Future prospective studies are needed to confirm this observation.

### Limitations

When reviewing the results of this meta-analysis, several limitations should be noted. Although our study included 4 RCTs, the remaining 12 studies were observational and therefore subject to selection bias. Consequently, we were unable to account for the inherent heterogeneity in the 2 types of study design (ie, inadequate blinding, lack of randomization due to the retrospective nature of the data, and patient preferences for individual management strategies). Our subgroup analysis according to study type demonstrated that pooled outcome estimates did not vary substantially from estimates solely derived from RCTs. The studies included in the meta-analysis also had variable follow-up periods. However, this potential limitation was accounted for by reporting outcomes as IRR. For studies reporting mean follow-up, the median follow-up was estimated assuming an exponential distribution; however, skew could not be assessed adequately because most studies failed to report the standard deviation of follow-up duration or provide a Kaplan-Meier curve for the time to study discontinuation. The 3 studies excluded from the primary analysis for reporting only full study length also did not report event rates for other outcomes of interest (cardiovascular mortality, HF hospitalization, or stroke)^11^^,^^14^^,^^17^; hence, the sensitivity analysis for all-cause mortality which included these 3 studies was the only one feasible because of data availability.

In addition, the absence of patient-level data at the time of analysis limited our ability to examine the source of higher heterogeneity for estimates of all-cause and cardiovascular mortality. Furthermore, hazard ratios were not reported for some observational studies because they did not have sufficient data granularity (eg, retrospective cohort studies with incomplete data sets and only reported event counts). To maximize the number of studies included in this meta-analysis, we therefore adopted the IRR approach. Although our meta-analysis addressed both SAVR and TAVR, the majority of studies included SAVR as the sole treatment modality, and our findings are not intended to influence the selection of the mode of procedure in individual patients with asymptomatic severe AS. Given the variation in trial designs and patient characteristics across the studies included in our analysis, we did not explore the impact of TAVR versus SAVR on early treatment outcomes. In a recent meta-analysis of the 4 randomized trials evaluating a strategy of early TAVR or SAVR versus routine CS, a subgroup analysis comparing SAVR and TAVR trials showed no significant differences between SAVR and TAVR studies for all outcomes, except for all-cause mortality.^53^ Future research should focus on exploring the impact of AVR modality on clinical outcomes. Finally, differences in the timing of AVR after diagnosis of severe asymptomatic AS may influence the anticipated benefit of timely AVR in this population.

## Conclusion

The results of this meta-analysis show that a strategy of timely AVR for patients with asymptomatic severe AS is associated with a significant reduction in the rates of all-cause mortality, cardiovascular mortality, and unplanned cardiovascular or HF hospitalization when compared to CS, despite considerable heterogeneity in pooled mortality outcomes. Furthermore, evidence from RCTs alone demonstrated that the same strategy was associated with a significant reduction in stroke rates. Although the benefit of all-cause and cardiovascular mortality initially observed in observational studies and early RCTs was less pronounced in the most recent RCT, this is likely to be a consequence of enrolling a cohort of older patients and mandated high-quality CS. Our findings suggest that a strategy of timely AVR may be preferable for many patients with asymptomatic severe AS.
