# Supplementary material for: Aortic Valve Replacement in Asymptomatic Severe Aortic Stenosis: A Systematic Review and Meta-Analysis
Source: J Soc Cardiovasc Angiogr Interv. 2025 May 2;4(7):103663. doi: 10.1016/j.jscai.2025.103663 (PMC12418467; doi:10.1016/j.jscai.2025.103663)
Supplement: Supplementary Material [file mmc1.pdf]

Supplementary Material

Table S1. PICOS framework.

	Inclusion Criteria
**Population**	Patients with asymptomatic severe or very severe AS
**Intervention**	AVR: either surgical AVR or transcatheter AVR
**Comparator**	Clinical surveillance
**Outcomes**	• Primary: all-cause mortality<br>• Secondary: cardiovascular mortality, unplanned cardiovascular or HF hospitalization, and stroke
**Study Design**	• RCTs<br>• Observational studies (prospective and retrospective)

AS, aortic stenosis; AVR, aortic valve replacement; HF, heart failure; RCTs, randomized controlled trials

Table S2. Search strategy.

Database	Time Period	Raw text string	MeSH terms
**PubMed**	Inception to November 11, 2024	asymptomatic AND severe AND "aortic stenosis" AND ("aortic valve replacement" OR SAVR OR TAVR OR TAVI OR "transcatheter aortic valve" OR "conservative management" OR "watchful waiting")	("asymptomatic"[All Fields] OR "asymptomatically"[All Fields] OR "asymptomatics"[All Fields]) AND ("sever"[All Fields] OR "severe"[All Fields] OR "severed"[All Fields] OR "severely"[All Fields] OR "severer"[All Fields] OR "severes"[All Fields] OR "severing"[All Fields] OR "severities"[All Fields] OR "severity"[All Fields] OR "severs"[All Fields]) AND "aortic stenosis"[All Fields] AND ("aortic valve replacement"[All Fields] OR "SAVR"[All Fields] OR "TAVR"[All Fields] OR "TAVI"[All Fields] OR "transcatheter aortic valve"[All Fields] OR "conservative management"[All Fields] OR "watchful waiting"[All Fields]) NOT (casereports[Filter] OR editorial[Filter] OR letter[Filter]
**EMBASE**			asymptomatic AND severe AND ('aortic stenosis'/exp OR 'aortic stenosis') AND ('aortic valve replacement'/exp OR 'aortic valve replacement' OR savr OR tavr OR 'tavi'/exp OR tavi OR 'transcatheter aortic valve'/exp OR 'transcatheter aortic valve' OR 'conservative management'/exp OR 'conservative management' OR 'watchful waiting'/exp OR 'watchful waiting') NOT ('editorial'/it OR 'letter'/it OR 'animal model'/de OR 'conference abstract'/it)
**Clinicaltrials.gov**			"asymptomatic severe aortic stenosis" in Condition/disease keyword AND "aortic valve replacement" OR "SAVR" OR "TAVR" OR "TAVI" OR "transcatheter aortic valve" OR "conservative management" OR "watchful waiting" in Other terms keyword (Word variations were searched)

Table S3. Risk of bias assessment- RoB2 Tool for RCTs.

Reference, year	Randomization Process	Deviation from the Intended Interventions	Missing Outcome Data	Measurement of the Outcome	Selection of the Reported Result	Overall Bias
Genereux et al,[27] 2025 EARLY TAVR	+	+	+	+	+	+
Loganath et al,[28] 2025 EVOLVED	+	+	+	+	+	+
Banovic et al,[26] 2024 AVATAR	+	+	+	+	+	+
Kang et al,[25] 2020 RECOVERY	+	+	+	+	+	+

Judgements of risk for each domain include low risk of bias (+), some concerns (!), or high risk of bias (-). RCT, randomized controlled trial.

Table S4. Risk of bias assessment- Newcastle-Ottawa Scale for observational studies.

Reference, year	Selection	Comparability	Outcome	TOTAL (max 9)
Çelik et al,[18] 2021	***	**	***	8
Campo et al,[17] 2019	***	**	***	8
Kim et al, [16] 2019	***	**	***	8
Bohbot et al, [15] 2018	***	-	***	6
Oterhals et al, [14] 2017	***	**	**	7
Masri et al, [13] 2016	***	**	**	7
Taniguchi et al, [12] 2015	***	**	***	8
Heuvelman et al, [11] 2012	***	**	***	8
Le Tourneau et al, [10] 2010	***	**	***	8
Kang et al, [9] 2010	***	**	***	8
Pai et al, [8] 2006	***	**	**	7
Pellikka et al, [7] 2005	***	**	***	8

The Newcastle-Ottawa Scale includes three indices for assessing study quality of non-randomized studies, including selection, comparability, and outcome. Each asterisk represents one point in each category on the Newcastle-Ottawa Scale; total scores range from 0 to 9 stars, with scores of 7 or more considered high quality and scores less than 7 considered low quality. The quality of the observational data was good; however, one study provided very little detail concerning baseline patient characteristics for the AVR and CS groups and was rated at 6 points with a low rating for comparability.

Study	AVR Events	AVR Time	CS Events	CS Time	Weight	IRR [95% CI]
Celik et al,[18] 2021	1	26.70	4	44.50	1.4%	0.42 [0.05; 3.73]
Kang et al,[25] 2020	5	452.60	15	439.20	4.5%	0.32 [0.12; 0.89]
Campo et al,[17] 2019	9	520.00	34	805.00	6.2%	0.41 [0.20; 0.85]
Kim et al,[16] 2019	37	1541.67	109	1197.80	9.0%	0.26 [0.18; 0.38]
Bohbot et al,[15] 2018	21	672.00	91	864.50	8.2%	0.30 [0.18; 0.48]
Oterhals et al,[14] 2017	1	10.50	4	36.00	1.4%	0.86 [0.10; 7.67]
Masri et al,[13] 2016	44	2352.90	60	1324.80	8.9%	0.41 [0.28; 0.61]
Taniguchi et al,[12] 2015	40	1084.33	69	1084.33	8.9%	0.58 [0.39; 0.86]
Heuvelman et al,[11] 2012	3	44.00	2	74.00	2.0%	2.52 [0.42; 15.10]
Le Tourneau et al,[10] 2010	31	906.11	181	2910.89	8.9%	0.55 [0.38; 0.81]
Kang et al,[9] 2010	3	353.60	28	460.75	3.7%	0.14 [0.04; 0.46]
Pai et al,[8] 2006	9	346.50	129	836.50	6.6%	0.17 [0.09; 0.33]
Pellikka et al,[7] 2005	41	783.00	103	972.00	9.1%	0.49 [0.34; 0.71]
Genereux et al,[27] 2025	38	1683.50	41	1694.80	8.5%	0.93 [0.60; 1.45]
Loganath et al,[28] 2025	16	395.50	14	388.50	6.3%	1.12 [0.55; 2.30]
Banovic et al,[26] 2024	11	409.50	27	414.75	6.4%	0.41 [0.20; 0.83]
**Total (95% CI)**					**100.0%**	**0.44 [0.33; 0.59]**

Heterogeneity: $Tau^2 = 0.1948$; $Chi^2 = 46.97$, df = 15 ($P < 0.01$); $I^2 = 68\%$
Test for overall effect: $Z = -5.63$ ($P < 0.01$)

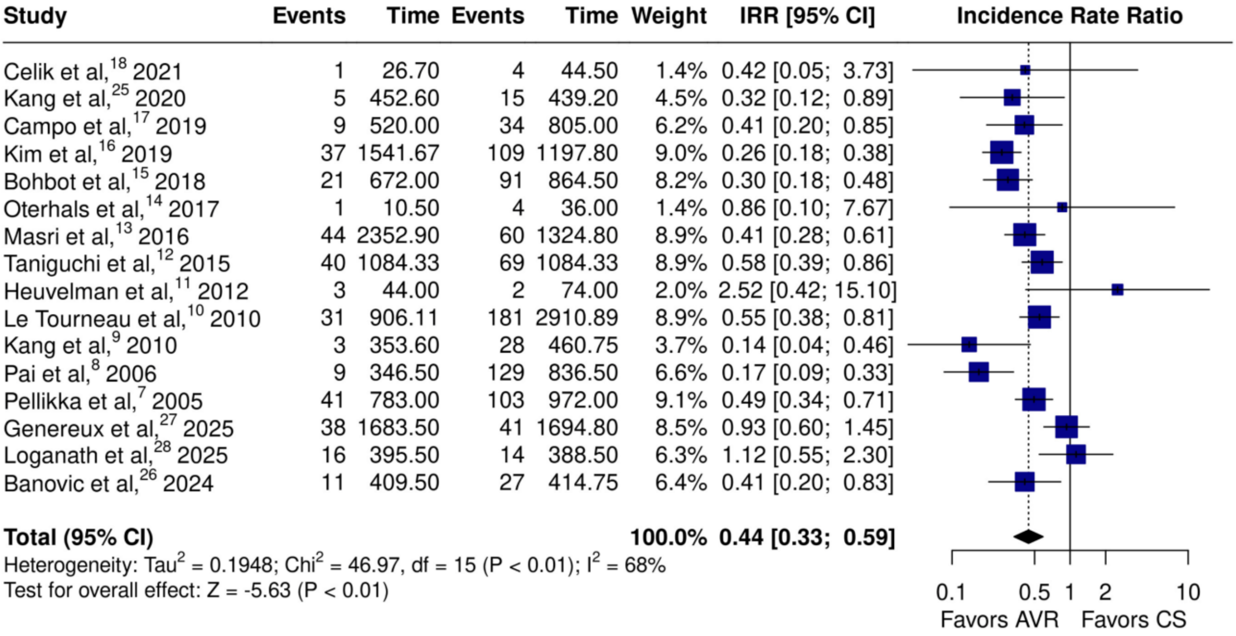

Figure S1. Meta-analysis of AVR versus CS comparing incidence rates of all-cause mortality, sensitivity analysis- all studies included.

AVR, aortic valve replacement; CI, confidence interval; CS, clinical surveillance; IRR, incidence rate ratio.

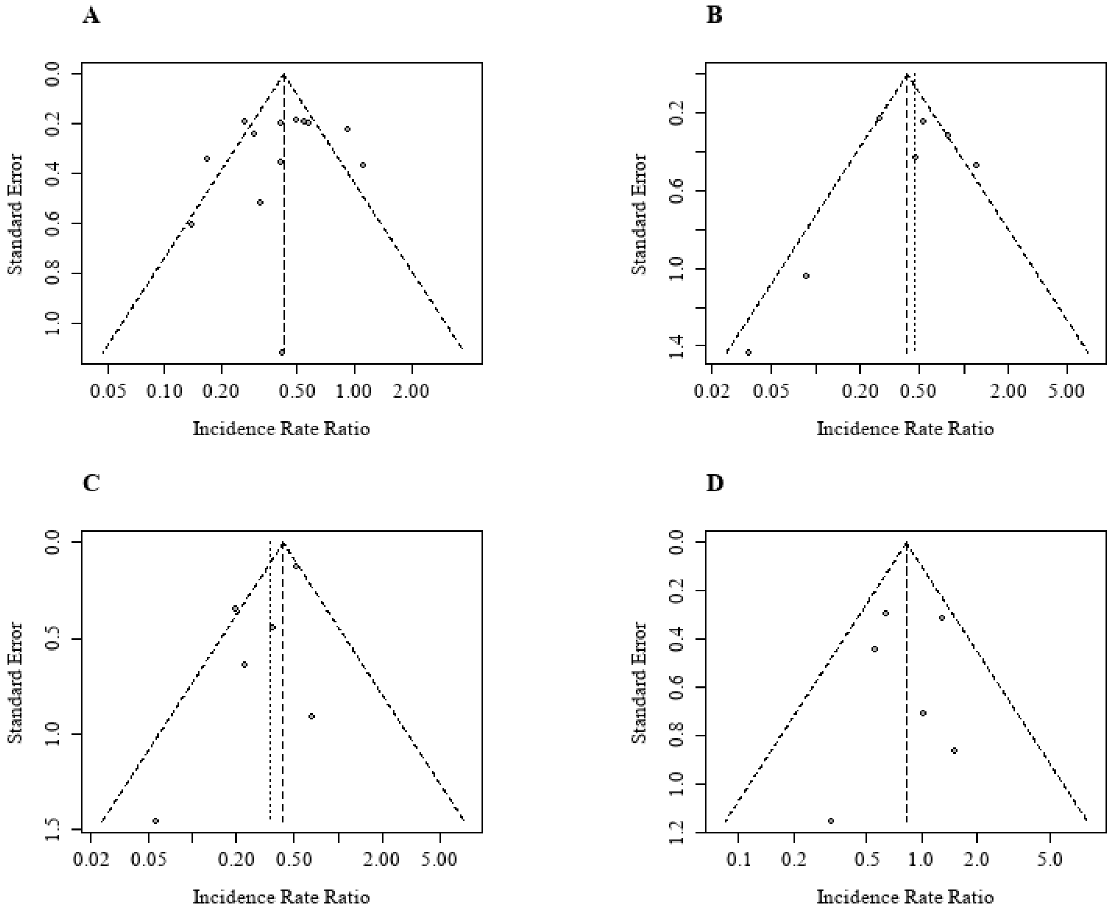

Figure S2. Funnel plots for all-cause mortality, cardiovascular mortality, unplanned cardiovascular or HF hospitalization, and stroke.

Funnel plots for assessment of publication bias for the incidence of **(A)** all-cause mortality, **(B)** cardiovascular mortality, **(C)** unplanned cardiovascular or HF hospitalization, and **(D)** stroke as derived from RCTs and observational studies.
HF, heart failure; RCT, randomized controlled trial.
